# BTK Inhibition Reverses MDSC-Mediated Immunosuppression and Enhances Response to Anti-PDL1 Therapy in Neuroblastoma

**DOI:** 10.3390/cancers13040817

**Published:** 2021-02-16

**Authors:** Mehreen Ishfaq, Timothy Pham, Cooper Beaman, Pablo Tamayo, Alice L. Yu, Shweta Joshi

**Affiliations:** 1 Division of Pediatric Hematology-Oncology, Moores Cancer Center, University of California, San Diego, CA 92093-0815, USA; mishfaq@health.ucsd.edu (M.I.); cooperbeaman@gmail.com (C.B.); 2 Office of Cancer Genomics, University of California, San Diego, CA 92093-0815, USA; tvp005@health.ucsd.edu (T.P.); ptamayo@ucsd.edu (P.T.); 3 Department of Pediatrics, University of California, San Diego, CA 92093-0815, USA; a1yu@health.ucsd.edu; 4 Institute of Stem Cell and Translational Cancer Research, Chang Gung Memorial Hospital at Linkou, Chang Gung University, Taoyuan 131, Taiwan

**Keywords:** myeloid derived suppressor cells, neuroblastoma, Bruton tyrosine kinase, immunosuppression

## Abstract

**Simple Summary:**

Neuroblastoma (NB) is the most common pediatric malignancy, and patients with the high-risk disease show a worse prognosis despite advanced treatments, including immunotherapy. Myeloid-derived suppressor cells (MDSC) frequently accumulate in NB tumors, where they induce immunosuppression and hamper efficient antitumor immune responses. In the current study, we observed that Bruton’s tyrosine kinase (BTK) is highly expressed in both monocytic and granulocytic MDSCs isolated from spleens of mice bearing NB tumors and administration of BTK inhibitor ibrutinib reduced MDSC-mediated immunosuppression, tumor growth, and enhanced anti-PDL1 checkpoint inhibitor therapy in mice bearing NB tumors. These studies demonstrated that ibrutinib could serve as a promising therapeutic agent to control MDSC-mediated immune suppression in NB.

**Abstract:**

MDSCs are immune cells of myeloid lineage that plays a key role in promoting tumor growth. The expansion of MDSCs in tumor-bearing hosts reduces the efficacy of checkpoint inhibitors and CAR-T therapies, and hence strategies that deplete or block the recruitment of MDSCs have shown benefit in improving responses to immunotherapy in various cancers, including NB. Ibrutinib, an irreversible molecular inhibitor of BTK, has been widely studied in B cell malignancies, and recently, this drug is repurposed for the treatment of solid tumors. Herein we report that BTK is highly expressed in both granulocytic and monocytic murine MDSCs isolated from mice bearing NB tumors, and its increased expression correlates with a poor relapse-free survival probability of NB patients. Moreover, in vitro treatment of murine MDSCs with ibrutinib altered NO production, decreased mRNA expression of *Ido*, *Arg*, *Tgfβ*, and displayed defects in T-cell suppression. Consistent with these findings, in vivo inhibition of BTK with ibrutinib resulted in reduced MDSC-mediated immune suppression, increased CD8+ T cell infiltration, decreased tumor growth, and improved response to anti-PDL1 checkpoint inhibitor therapy in a murine model of NB. These results demonstrate that ibrutinib modulates immunosuppressive functions of MDSC and can be used either alone or in combination with immunotherapy for augmenting antitumor immune responses in NB.

## 1. Introduction

Neuroblastoma (NB) is an extremely heterogeneous pediatric solid tumor that develops from immature nerve cells of the sympathetic nervous system. These extracranial tumors commonly arise in and around the adrenal glands and are rare in kids older than 10 years of age [1,2] The amplification of MYCN oncogene is frequently observed in NB tumors and despite intensive multimodal treatments, which include chemotherapy, radiotherapy, anti-GD2 immunotherapy, and stem cell transplant, the overall survival of patients diagnosed with high-risk disease is less than 50%. The infiltration and accumulation of immunosuppressive myeloid cells, specifically myeloid-derived suppressor cells (MDSC) and tumor-associated macrophages (TAM) in NB tumors, diminish effective antitumor immune responses, which leads to failure of immunotherapy and other conventional therapies in this childhood cancer [3,4,5].

MDSCs are immature myeloid cells that play a pivotal role in promoting tumor growth and making tumors resistant to immunotherapy and other conventional therapies by employing several mechanisms, which includes secretion of immunosuppressive cytokines, generation of regulatory T lymphocytes (Tregs) and production of nitric oxide (NO) and reactive oxygen species (ROS) [6,7]. Based on the expression of Ly6G and Ly6C, MDSC is classified into two subsets in mice: monocytic MDSC (M-MDSC), characterized as CD11b+Ly6G^-^Ly6C^high^, and granulocytic MDSC (G-MDSC) characterized as CD11b+Ly6G^high^Ly6C^low^. Both subsets of MDSCs utilize diverse mechanisms to hinder the antitumor activity of T cells, which leads to uncontrolled tumor growth [8]. Recent preclinical studies have shown that strategies aimed at inhibiting the immunosuppressive functions of MDSCs can improve the efficacy of checkpoint inhibitors and CAR-T therapies in this childhood cancer [9,10,11,12]. Hence, finding novel and effective approaches to deplete or inhibit the function of these immunosuppressive myeloid cells may provide benefit to treat this childhood malignancy.

Ibrutinib, an FDA-approved inhibitor of Bruton’s tyrosine kinase (BTK), is highly effective in the treatment of B cell malignancies [13]. Beyond its role in B-cell signaling, BTK plays an important role in the development and function of myeloid cells [14,15,16]. Recent studies have shown that BTK is present in human and murine MDSCs and administration of ibrutinib blocks MDSC-mediated immunosuppression in melanoma and breast carcinoma tumor models [17,18]. Ibrutinib also inhibits ITK, an enzyme important for the development of Th2 cells, and by doing so, it stimulates Th1 responses and enhances antitumor immunity in solid tumors [17,18,19]. The expression of BTK in neuroblastoma cell lines and tumor tissues has been recently reported, but its role in the immune microenvironment and MDSC of NB are unknown [20].

Herein, we report that BTK is expressed in both M-MDSC and G-MDSC isolated from mice bearing NB tumors and inhibiting BTK with ibrutinib significantly decreased the expansion of MDSC in vivo and boosted the efficacy of checkpoint blockade in NB. These studies open new avenues to combine ibrutinib with other immune-based therapies for the treatment of neuroblastoma.

## 2. Results

### 2.1. High Expression of Myeloid BTK Correlates with Poor Survival in Neuroblastoma Patients

**Figure 1:**
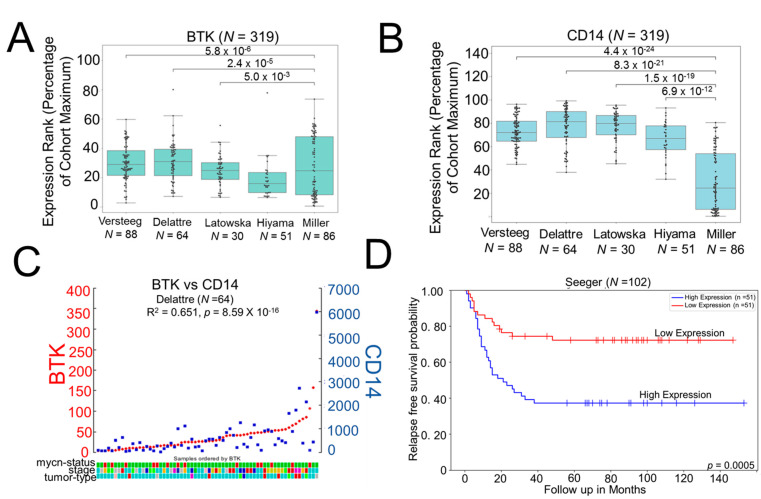
Bruton’s tyrosine kinase (BTK) is a prognostic factor in neuroblastoma (NB): (**A**,**B**) Gene expression of BTK and CD14 was analyzed in different neuroblastoma cohorts, Versteeg *N* = 88; Delattre *N* = 64; Latowska *N* = 30; and Hiyama *N* = 51 and benign neurofibroma (Miller, *N* = 86) using the R2 genomics database. Data were analyzed using one-way ANOVA; all values are *p* ≤ 0.001 compared to Miller (*n* = 86), neurofibroma). (**C**) Positive correlation between BTK and CD14 expression in Delattre cohort. (**D**) Prognostic significance of intramural expression of BTK in non-MYCN-amplified Seeger dataset of NB (*p* = 0.0005).

To evaluate if myeloid BTK can predict survival in human NB, we used publicly available R2 Genomics and Visualization platform (http://r2.amc.nl, accessed on 16 February 2021) and analyzed the expression of BTK and monocyte marker CD14 in four different datasets (cohort 1, Versteeg *N* = 88; cohort 2, Delattre *N* = 64; cohort 3, Latowska *N* = 30; cohort 4, Hiyama *N* = 51). We found that the expression of both BTK and monocyte marker CD14 is elevated in various neuroblastoma cohorts compared to benign neurofibroma (Miller, *N* = 86) (Figure 1A,B). We next evaluated if there is any correlation between the expression of BTK and CD14 in NB cohorts. Interestingly, expression of CD14 correlated strongly with the expression of BTK in benign neurofibroma (Miller, *N* = 86) as well as in different NB cohorts except for Hiyama (Figure S1 and Figure 1C). Furthermore, we examined the prognostic significance of BTK expression and found that increased expression of BTK significantly correlates with worse overall survival (OS) in the non-MYCN amplified neuroblastoma cohort (Seeger dataset) (Figure 1D).

### 2.2. BTK Is Expressed in MDSC Isolated from Mice Bearing Neuroblastoma Tumors

**Figure 2:**
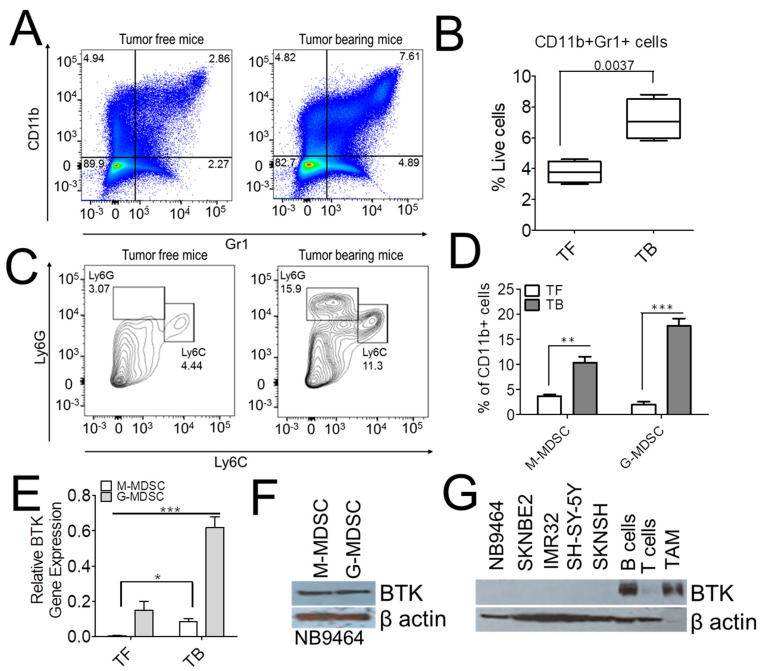
BTK is expressed in both M-MDSC and G-MDSC isolated from mice bearing neuroblastoma tumors. (**A**,**B**) Increase in the infiltration of MDSCs in mice bearing neuroblastoma tumors. 4 × 10^6^ NB9464 cells were injected in the flank regions of C57Bl/6 mice, and spleens were harvested after 35 days of tumor inoculation. Single-cells obtained from the spleens of tumor-free (TF) and mice bearing NB9464 tumors (TB) (*n* = 5 mice/group) were stained with antibodies against CD11b and Gr-1 and analyzed by flow cytometry. Figure 2A,B shows the percentage of CD11b^+^/Gr-1^+^ cells in the spleens from TF and TB mice (Cells were gated on live cells). *p*-value was calculated using an unpaired t-test. (**C**,**D**) Figure shows the graphic (**C**) and quantitative (**D**) representation of the number of G-MDSC and M-MDSC subsets in the spleens of TF and TB mice (Cells were gated on CD11b+ cells). (**E**,**F**) mRNA (**E**) and protein (**F**) expression of BTK in M-MDSC and G-MDSC isolated from the spleen of TF and TB mice. (**G**) Protein expression of BTK in murine and human MYCN amplified cell lines, NB9464, SKNBE2, IMR32; human MYCN-non-amplified cell lines, SH-SY-5Y and SKN-SH; CD19+ B cells, CD90.2+ T cells isolated from spleens of TB mice and CD11b+F4/80+ TAMs isolated from NB9464 tumors implanted in C57Bl/6 mice. Data in D and E were analyzed by 2-way ANOVA using Bonferroni posttests, * *p* ≤ 0.05, ** *p* ≤ 0.01, *** *p* ≤ 0.001.

BTK regulates the development and function of myeloid cells [14,15,16]; hence we investigated their role in MDSC. To test this, NB9464 cells were injected subcutaneously in C57Bl/6 mice, and MDSC subsets were characterized in spleens of naïve mice or tumor-free mice (TF) and mice bearing neuroblastoma tumors (TB). We found that NB-tumor-bearing (TB) mice show higher percentages of CD11b+Gr1+ cells in the spleen as compared to tumor-free (TF) mice (Figure 2A,B). We next investigated the presence of granulocytic MDSC (G-MDSC) and monocytic MDSC (M-MDSC) subsets in the spleens of TF and TB mice. Based on the differential expression of Ly6G and Ly6C, we found that the number of G-MDSC and M-MDSC isolated from TB mice is higher than TF mice (Figure 2C,D). Interestingly, we found that BTK was highly expressed in both M-MDSC as well as G-MDSC isolated from TB mice as compared to TF mice. (Figure 2E). However, mRNA expression analysis showed higher expression of BTK in G-MDSC as compared to M-MDSC in TB mice (Figure 2E), but there is no difference in the protein expression of BTK in M-MDSC and G-MDSC isolated from TB mice (Figure 2F). We next investigated the presence of BTK in NB cell lines as well as in other immune cells infiltrated in the NB TME. Li et al. have recently shown that BTK is expressed in neuroblastoma cell lines, but contrary to this report [20], we did not observe the expression of BTK in MYCN amplified (SKNBE2, IMR32) and non-MYCN amplified (SH-SY-5Y, SKNSH) human neuroblastoma cell lines (Figure 2F). We also did not observe the expression of BTK in murine NB cell line NB9464 used in the current study. Interestingly, we find higher expression of BTK only in B cells, tumor-associated macrophages (TAMs), and MDSC isolated from neuroblastoma tumors (Figure 2E,G).

### 2.3. Ibrutinib Inhibits MDSC NO Production and Immunosuppressive Functions in NB

**Figure 3:**
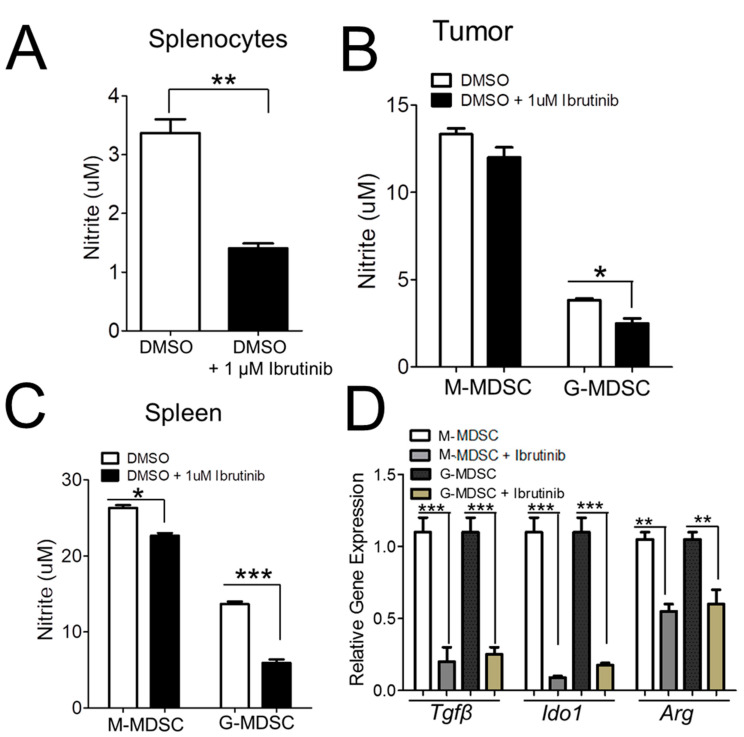
Ibrutinib reduces the production of nitric oxide and modulates the immunosuppressive functions of MDSCs in NB: (**A**) Effect of ibrutinib on the production of NO. Single-cell suspension prepared from the spleens of mice bearing NB9464 tumors were treated with 1 µM ibrutinib followed by stimulation with 1 µg/mL LPS and 20 ng/mL IFNγ. Nitrite levels were measured in the supernatants after 24 h of incubation using Griess reagent. *p*-value was calculated using an unpaired *t*-test, ** *p* ≤ 0.01. (**B**,**C**) Nitrite levels were measured in ibrutinib-treated M-MDSCs and G-MDSCs isolated from NB9464 tumors (**B**) and spleens (**C**) of mice bearing NB9464 tumors. (**D**) mRNA expression of *Tgfβ*, *Ido1*, and *Arg* in ibrutinib-treated M-MDSCs and G-MDSCs isolated from spleens of mice bearing NB9464 tumors and stimulated with 10 ng/mL IL6 and GMCSF. RNA was isolated after 24 h, and the expression of genes was determined by qRT–PCR. Data were analyzed (**B**–**D**) by 2-way ANOVA using Bonferroni posttests, * *p* ≤ 0.05, ** *p* ≤ 0.01, *** *p* ≤ 0.001.

MDSCs mediate immunosuppression by producing nitric oxide (NO) and increasing the secretion of Arginase (Arg), indoleamine 2, 3-dioxygenase (IDO), and transforming growth factor (TGFβ) to inhibit effective immune responses [21]. Hence, we first investigated if BTK inhibitor, ibrutinib, can reduce the production of NO in the splenocytes of MDSCs isolated from the NB9464 tumors or spleens of mice bearing NB9464 tumors. For this, M-MDSCs and G-MDSCs were isolated using a murine MDSC isolation kit. We found that treatment of lipopolysaccharide (LPS)-stimulated splenocytes with ibrutinib at a concentration of 1 µM decreased the production of NO (Figure 3A). Similarly, ibrutinib-treated M-MDSCs and G-MDSCs isolated from tumors or spleens of tumor-bearing mice lead to a significant decrease in NO production, with the pronounced effect of ibrutinib on G-MDSCs (Figure 3B,C).

We next studied if ibrutinib can reduce the mRNA expression of *Arg*, *Ido1,* and *Tgfβ* in M-MDSCs and G-MDSCs. For this, M-MDSCs and G-MDSCs isolated from NB-tumor-bearing mice were stimulated with DMSO or ibrutinib for 1 h, followed by the addition of IL6 and GMCSF for 24 h and analysis of mRNA expression of *Arg*, *Ido1,* and *Tgfβ* by qRTPCR. Treatment of both M-MDSCs and G-MDSCs with ibrutinib decreased the expression of these immunosuppressive cytokines (Figure 3D).

### 2.4. Ibrutinib Reduces MDSC-Mediated T-Cell Suppression in NB

**Figure 4:**
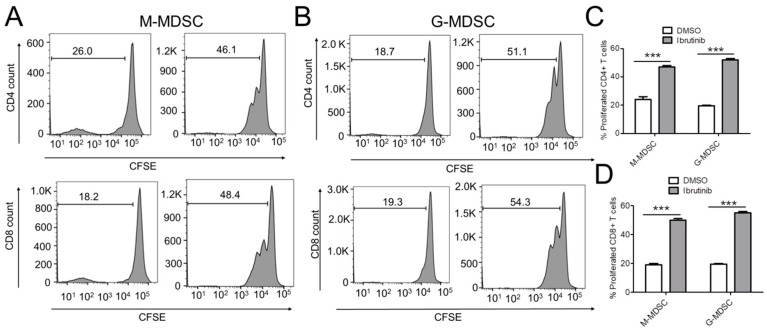
Ibrutinib reduces both M-MDSC and G-MDSC-mediated T-cell suppression in NB. (**A**–**D**) T cells isolated from naïve mice were labeled with CFSE and were allowed to adhere on plates coated with anti-CD3 and anti-CD28 antibodies. After 24 h of incubation, CFSE-labeled T cells were cultured with DMSO or ibrutinib-treated M-MDSCs and G-MDSCs isolated from spleens of NB tumor-bearing mice at a ratio of 2:1. After two days of culture, cells were analyzed for expression of CD4 and CD8 by flow cytometry. Histograms represent data from only one experiment, while bar graphs shown in (**C**,**D**) represent data from 2 independent experiments. Data were analyzed by 2-way ANOVA using Bonferroni posttests, *** *p* ≤ 0.001.

To determine if ibrutinib can relieve MDSC-mediated suppression on T cell activation and can increase the proliferation of T cells, we performed a carboxyfluorescein succinimidyl ester (CFSE) dilution assay. For this, T cells isolated from naïve mice were labeled with CFSE and were plated on CD3/CD28-coated plates. The next day, M-MDSCs and G-MDSCs isolated from mice bearing NB9464 tumors were treated with ibrutinib and were co-cultured with CFSE-labeled T cells for another 48 h, followed by flow cytometry analysis of proliferated CD4+ and CD8+ T cells. We found that ibrutinib-treated M-MDSCs and G-MDSCs both showed increased proliferation of CD4+ and CD8+ T cells as compared to DMSO-treated MDSCs (Figure 4A–D). These results suggest that ibrutinib decreases the immunosuppressive functions of MDSC with a greater influence on G-MDSCs than M-MDSCs.

### 2.5. Ibrutinib Suppresses Tumor Growth and Infiltration of MDSCs in Neuroblastoma

**Figure 5:**
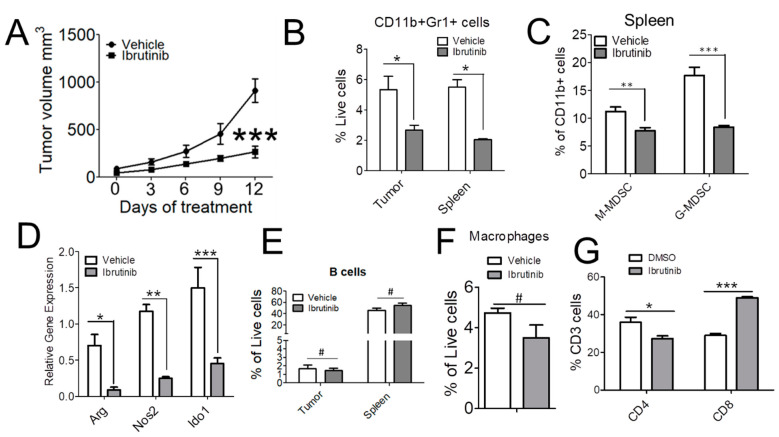
Ibrutinib reduces tumor growth and restricts infiltration of both M-MDSCs and G-MDSCs in NB. (**A**) Tumor growth of NB9464 tumors was significantly impaired in C57BL/6 WT (*N* = 7) mice treated with 25 mg/kg ibrutinib (5 days/week). (**B**,**C**) Flow cytometry quantification of CD11b+Gr1+ MDSC (cells were gated on live cells) (**B**) and M-MDSC and G-MDSC (**C**) (cells were gated on CD11b+ cells) isolated from tumors and spleens of mice bearing NB9464 tumors treated with 25 mg/kg ibrutinib, (*N* = 3). (**D**) mRNA expression of *Arg*, *Nos2* and *Ido1* was analyzed in MDSC isolated from spleens of vehicle and ibrutinib-treated mice bearing NB9464 tumors. (**E**–**G**) Flow cytometry quantification of different immune cells like CD19+B220+ B cells (**E**), CD11b+F4/80+ macrophages (**F**) (cells were gated on live cells), and CD4+ CD8+ T cells (gated on CD3+ T cells) (**G**) isolated from NB9464 tumors implanted in C57Bl/6 mice and treated with ibrutinib (*n* = 5), *t*-test was performed on (**E**,**F**), #, not significant, compared to vehicle. Data were analyzed by 2-way ANOVA using Bonferroni posttests in (**A**–**D**,**G**), * *p* ≤ 0.05; ** *p* ≤ 0.01; *** *p* ≤ 0.001, compared to vehicle.

We next investigated if ibrutinib can reduce tumor growth in MYCN amplified neuroblastoma tumor model. C57Bl/6 mice bearing NB9464 neuroblastoma tumors were treated with ibrutinib (25 mg/kg) or vehicle five times a week until tumors and spleens were harvested. Ibrutinib-treatment resulted in a significant reduction in tumor growth (Figure 5A), as well as in the number of CD11b+Gr1+ MDSCs infiltrated in the tumors and spleens of mice treated with ibrutinib (Figure 5B). We found that ibrutinib-treatment significantly reduced both the M-MDSC and G-MDSC populations isolated from the spleens of mice bearing NB tumors (Figure 5C). Furthermore, the treatment of tumors with ibrutinib reduced the mRNA expression of *Arg*, *Nos2*, and *Ido1* in MDSCs isolated from spleens of mice bearing NB tumors (Figure 5D). We found that BTK is also expressed in B cells as well as in TAMs isolated from mice bearing neuroblastoma tumors (Figure 2G). Hence, we next investigated if treatment of mice with ibrutinib has any effect on the infiltration of B cells or TAMs in the tumors. Importantly, we did not see any difference in the frequency of CD19+B220+ B cells (Figure 5E and Figure S2) or CD11b+F4/80+ TAMs in spleens or tumors of mice treated with ibrutinib (Figure 5F and Figure S3). Interestingly we find a significant decrease in the infiltration of CD4+ T cells and a significant increase in the infiltration of CD8+T cells in the tumors treated with ibrutinib (Figure 5G). Most notably, we did not see any decrease in the proliferation of either murine NB9464 cells or MYCN and non-MYCN amplified human NB cell lines treated with ibrutinib in vitro (Figure S4). Altogether, these results suggest that ibrutinib inhibits tumor growth by depleting MDSCs from NB tumors.

### 2.6. Ibrutinib Augmented Anti-PDL1 Therapy in Neuroblastoma

**Figure 6:**
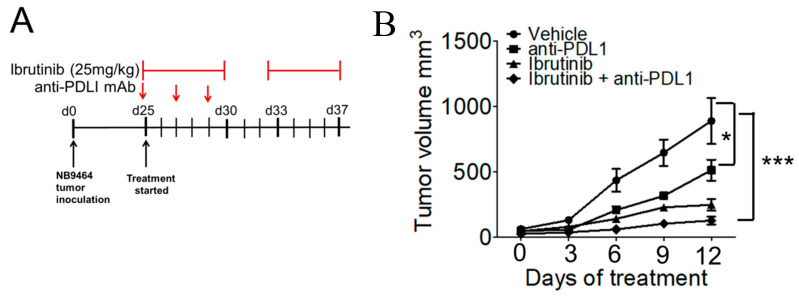
Ibrutinib augmented anti-PDL1 immune checkpoint blockade in neuroblastoma. (**A**) Schematic showing tumor implantation and treatment strategy in murine NB9464 tumors. (**B**) Tumor volume of NB9464 tumors treated with 25 mg/kg ibrutinib (5 times/week) alone or in combination with 100 μg anti-PDL1 mAb (3 doses) (*n* = 5), 2-way ANOVA using Bonferroni posttests, * *p* ≤ 0.05, *** *p* ≤ 0.001.

The combination of small molecule inhibitors with immune checkpoint blocking antibodies has shown efficacy in enhancing antitumor immune responses in various solid tumors [22]. Given the fact that strategies that reduce MDSC-mediated immunosuppression can enhance the efficacy of checkpoint blockade in various tumors, we investigated if treatment of ibrutinib can improve the anti-PDL1 therapy in NB. For this, mice bearing NB9464 tumors were randomized in four different groups when tumors reached 100 mm^3^ and were treated with either vehicle (IgG antibody), or 100 µg anti-PDL1 mAb or 25 mg/kg ibrutinib or the combination of ibrutinib and anti-PDL1mAb as shown in Figure 6A. The combination of ibrutinib and anti-PDL1 mAb significantly reduced tumor growth as compared to the use of a single agent (Figure 6B). These results suggest that ibrutinib can potentially synergize with checkpoint blockade in NB and can provide benefit to patients who do not show any benefit with checkpoint inhibitors as monotherapy.

## 3. Discussion

Immune checkpoint blockade alone has not shown any significant benefit in the treatment of NB. However, strategies that block myeloid-mediated immunosuppression are able to subvert the immune tolerance in this childhood cancer [10,23]. Hence, safe and efficacious myeloid-targeted agents are needed, which can be combined with immunotherapy to improve the survival of patients suffering from this disease.

BTK plays a crucial role in B-cell signaling, and recently, its role in innate immune cells has been recognized [17,24]. Ibrutinib, an irreversible inhibitor of BTK and ITK, has recently been shown to enhance antitumor immunity when used in combination with checkpoint blockade [18,19]. Here, we report that BTK is expressed in both monocytic and granulocytic MDSCs isolated from the murine NB tumors. Ibrutinib reduced NO production, mRNA expression of immunosuppressive cytokines and T-cell suppression mediated by both monocytic and granulocytic MDSCs of NB. Using a mouse model of neuroblastoma, we have shown that ibrutinib-treatment resulted in a significant reduction in tumor growth and number of MDSCs in vivo. Finally, ibrutinib was able to enhance the efficacy of anti-PDL1 immune checkpoint blockade in NB.

The role of BTK in neuroblastoma tumorigenesis has been reported recently [20]. This report has shown that BTK potentiates ALK-mediated signaling in neuroblastoma tumors, and combined treatment of ibrutinib with ALK inhibitor crizotinib can reduce the tumor growth of NB xenografts in nude mice. Consistent with this study, we find higher expression of BTK in NB patient cohorts, but contrary to this study, we did not find expression of BTK in any MYCN amplified or non-MYCN amplified human cell-lines. Li et al. have shown the expression of BTK in NBL and SH-SY-5Y cells, with higher expression of BTK in NBL cells. In this study, we have used SKNBE2, IMR32, SKNSH, and SH-SY-5Y cell lines and did not observe the expression of BTK in these cells. Consistent with these results, we also did not see any effect of ibrutinib on the proliferation of either murine or human neuroblastoma cell lines (Figure S4A). Our study has shown that BTK is mainly expressed in MDSCs, B cells, and TAMs isolated from mice bearing neuroblastoma tumors. Several reports have demonstrated the ability of ibrutinib to modulate the function of immune cells, including MDSCs, monocytes, and T cells, indicating that ibrutinib has a role in immunomodulatory responses [17,19]. Hence, the current study is focused on studying if BTK is expressed in MDSCs isolated from NB tumors and if ibrutinib can reduce MDSC-mediated immunosuppression to enhance the efficacy of anti-PDL1 therapy in NB.

Ibrutinib has recently been reported to demonstrate its ability to inhibit the generation of MDSCs [17]. This study has shown that BTK is expressed in MDSCs isolated from tumor-bearing hosts, and ibrutinib alters the functions of MDSCs, including NO production and migration. The authors found that treatment of monocytic cell line MSC2 or in vitro generated human MDSCs with ibrutinib have no effect on mRNA expression of *Nos2* and *Arg*. We found that BTK is expressed in both monocytic and granulocytic MDSCs isolated from the NB tumor model. We found a higher expression of BTK in both subsets of MDSCs from NB with higher mRNA expression of BTK in G-MDSCs as compared to M-MDSCs (Figure 2E). We found that ibrutinib is able to modulate the immunosuppressive functions of both populations of MDSCs in NB tumors (Figure 3 and Figure 4). MDSCs suppress T-cell activity through iNOS, arginase, IDO, and TGFβ activity. Our data have demonstrated that ibrutinib downregulated the expression of *Arg*, *Nos2*, and *Tgfβ* in MDSCs isolated from mice bearing NB tumors can block the production of NO (Figure 3). In addition, we found the reduced frequency of MDSCs infiltrated in ibrutinib-treated tumors (Figure 5). These results suggest that ibrutinib depletes MDSCs as well as modulates the immunosuppressive functions of MDSCs to suppress tumor growth and enhance responses to checkpoint blockade in NB.

## 4. Materials and Methods

### 4.1. R2 Genomics

The expression of BTK and monocyte marker CD14 was investigated in neuroblastoma patient datasets by using R2: a web-based genomics and visualization platform (http://r2.ac.nl, accessed on 16 February 2021). The expression data from four neuroblastoma patient datasets: Versteeg [25] (cohort 1, *N* = 88), Delattre [26] (cohort 2, *N* = 64) Hiyama [27] (cohort 3, *N* = 51) and Latowska [28] (cohort 4, *N* = 30) was compared with neurofibroma [29] (Miller, *N* = 86) using one-way ANOVA. The prognostic significance of intramural expression of BTK was evaluated in non-MYCN amplified Seeger data set (*N* = 102) using the R2 database [30].

### 4.2. Cell Lines

SKNBE2, IMR32, SKNSH, SH-SY-5Y cells were cultured in Dulbecco’s modified Eagle’s medium (DMEM) supplemented with 10% FBS (Gemini Bio, Sacramento, CA, USA), penicillin/streptomycin (100 U/mL, Thermo Fisher Scientific, San Diego, USA), L-glutamine (2 mM, Thermo Scientific, San Diego, CA, USA), sodium pyruvate (0.4 mM, Sigma-Aldrich, St. Louis, MO, USA), and non-essential amino acids (Thermo Fisher, San Diego, CA, USA) as described before [31,32]. SKNSH cells were generously provided by Alice Yu (University of California, San Diego, USA). Murine neuroblastoma cell line NB9464 was maintained in DMEM supplemented with 10% FBS, 100 U/mL penicillin/streptomycin and 5% M3 base medium as described before [33]. NB9464D cells derived from spontaneous NB tumors arising in TH-MYCN transgenic mice were obtained from Jon Wigginton [34]. All cell lines were routinely tested in our lab for mycoplasma contamination (MycoAlert, Lonza, Basel, Switzerland).

### 4.3. In Vivo Tumor Growth Experiments

All animal experiments were performed with approval from UCSD Animal Care Committee. Six-eight week old C57BL/6 mice used in these experiments were obtained from the breeding colony in our lab at UCSD. 4 × 10^6^ NB9464 neuroblastoma cells were injected intradermally in the dorsal flank of C57Bl/6 mice. Tumor dimensions were recorded regularly once when tumors were palpable using the following formula: Volume = 0.5 × length × (width)^2^. When tumors reached 100 mm^3^, mice were randomized and treated with ibrutinib or vehicle at a dose of 25 mg/kg (administered by oral gavage), five times a week until tumors were harvested. Ibrutinib was obtained from LC laboratories and was formulated in Formulation 3 of the Hot Red Chemistry Formulation Kit (Pharmatek Laboratories, San Diego, CA, USA). In another experiment, NB9464 cells were injected in C57BL/6 WT mice, and when tumors reached 100 mm^3^, mice were treated with 25 mg/kg ibrutinib (oral gavage, five days/week) either alone or in combination with 100 μg anti-PDL1 mAb (clone 10F.9G2, BioXCell, Lebanon, NH, USA) or isotype control LTF2 (Bio X cell) (injected intraperitoneally, 3 doses, Monday, Wednesday, and Friday, starting day 1 of ibrutinib-treatment).

### 4.4. Isolation of Single Cells from Tumors and Spleens and Flow Cytometry

Dissociation of tumors and spleens into single-cell suspensions and flow cytometry analysis of immune cells was performed as described before [35]. Briefly, tumors were enzymatically dissociated in Hanks balanced salt solution containing 0.5 mg/mL collagenase IV (Sigma), 0.1 mg/mL hyaluronidase V (Sigma), 0.6 U/mL Dispase II (Roche Diagnostics, IN, USA) and 0.005 MU/mL DNAse I (Sigma) at 37 °C for 30–45 min. Single-cell suspensions were filtered through a 70 μm cell strainer followed by red blood cell lysis using RBC lysis buffer (Pharm Lyse, BD Biosciences, San Jose, CA, USA). Spleens were minced and filtered through a 40 um cell strainer (BD biosciences). For flow cytometry staining, dissociated cells were incubated with Aqua live dead fixable stain (Life Technologies, Carlsbad, CA, USA) followed by anti-mouse CD16/CD32 Fc block (BD Biosciences). Primary antibodies to cell surface markers directed against CD45 (30-F11), CD11b (M1/70), Gr1 (RB6-8C5), Ly6G (clone 1A8), Ly6C (clone AL21), F4/80 (BM8), anti-Gr-1 (clone RB6-8C5), CD19 (ID3), B220 (RA3-6B2) were obtained from BD biosciences. For T cell analysis, single cells were incubated with CD3 (145-2C11), CD4 (GK1.5), CD8 (53–6.7) from eBioscience, followed by near IR live dead stain (Life Technologies). Samples were run on BD Canto RUO 11 color analyzer. Data were analyzed using FlowJo software version 10 (Treestar, Ashland, OR, USA).

### 4.5. Isolation of MDSC from NB Tumor-Bearing Mice

4 × 10^6^ NB9464 cells were implanted in C57BL/6 mice, and when tumors reached 1000 mm^3^, spleens were collected aseptically from mice. MDSC were purified using a murine myeloid-derived suppressor cell isolation kit (Miltenyi Biotec, Auburn, CA, USA). Briefly, CD11b+ cells were isolated from splenocytes of tumor-bearing mice using CD11b-conjugated microbeads followed by the use of murine myeloid-derived suppressor cell isolation kit (Miltenyi Biotec) to separate M-MDSC (Gr-1^dim^Ly6G−) and G-MDSCs (Gr1^high^Ly6G+) according to manufacturer’s instructions. To purify the Ly6G+ population, CD11b+ cells were magnetically labeled indirectly with biotin coupled-LY6G followed by staining with anti-biotin microbeads according to the manufacturer’s instructions. Ly6G− cells were further purified from the same spleen using biotin coupled-anti-Gr1 and streptavidin microbeads according to the manufacturer’s instructions.

### 4.6. Nitric Oxide Estimation

Splenocytes or MDSC isolated from spleens of tumor-bearing mice were treated with DMSO or 1 µM ibrutinib for 1 h followed by washing and plating of cells in triplicates in 96-well plates. For splenocytes, cells were seeded at a concentration of 3 × 10^5^ per well followed by stimulation with 1 µg/mL LPS and 20 ng/mL IFNγ for 24 h as previously described. In a separate experiment, CD11b+Gr1+ MDSC and CD11b+Gr1-MDSC were cultured with 100 ng/mL IL6 (Miltenyi Biotec) and 10 ng/mL GMCSF (Miltenyi Biotec) for 48 h. Nitrite levels were measured in these samples using Greiss reagent as described before [33].

### 4.7. In Vitro Suppressive Assay

M-MDSC and G-MDSC were isolated from mice bearing NB9464 tumors as described above. CD90.2 T cells were isolated from the spleen of naïve mice using CD90.2 microbeads beads (Miltenyi Biotec) according to the manufacturer’s instructions. For in vitro suppression assay, CellTrace^TM^ cell proliferation kit (Thermo Fisher Scientific) was used to label CD90.2 T cells with carboxyfluorescein succinimidyl ester (CFSE). CFSE-labeled cells were plated on 96-well plates precoated with 10 µg/mL soluble anti-CD3 and 10 µg/mL of anti-CD28 antibodies. In order to activate T cells, 10 ng/mL IL2 was added to CFSE-labeled T cells. After 24 h, M-MDSC and G-MDSC treated with 1 µM ibrutinib were co-cultured with CFSE-labeled T cells in different ratios in the presence of 10 ng/mL IL6 and 10 ng/mL GMCSF. After 48 h of co-culture, samples were stained with antibodies against CD4 and CD8 and proliferation of CD4+ and CD8+ T cells were accessed by flow cytometry.

### 4.8. Western Blotting

Human neuroblastoma cell lines, CD19+ B cells, CD90.2 T cells, CD11b+F4/80+ TAMs, CD11b+Gr1+ and CD11b+Gr1− MDSC were lysed in RIPA buffer, and lysates were subjected to Western blotting using antibodies against total BTK or GAPDH as described before.

### 4.9. RNA Extraction and Real-Time PCR

For quantitative RTPCR, total RNA was isolated from ibrutinib-treated MDSC using the Qiagen RNAeasy kit (Life Technologies). cDNA was prepared using 1 µg RNA with the iscript cDNA synthesis kit (Bio-Rad, Hercules, CA, USA). SYBR green-based qPCR was performed to measure the expression of *Arg1*, *Ido1* and *Nos2* using predesigned mouse primers (Integrated DNA Technologies) as described before [34]. The qPCR assays were run using the Bio-Rad CFX96 real-time PCR system. mRNA levels were normalized to *Gapdh*, and data are represented as relative mRNA expression or fold change.

### 4.10. Cell Viability Assay

Cell proliferation assay was performed on ibrutinib-treated SKNBE2, IMR32, SKNSH, SH-SY-5Yand NB9464 cells using alamarBlue^®^ (Roche) reagent according to the manufacturer’s protocol. Briefly, 1 × 10^4^ cells were seeded in 96-well plates, followed by the addition of ibrutinib at different concentrations. AlamarBlue^®^ was added after 48 h, and plates were incubated for another 4 h at 37 °C in 5% CO_2_. Changes in cell viability are detected using Tecan Infinite F200 proplate reader (Tecan, Mannedorf, Switzerland) and fluorescence signals were read as emission at 590 nm after excitation at 560 nm.

## 5. Conclusions

In conclusion, this report demonstrates that BTK is highly expressed in both M-MDSCs and G-MDSCs isolated from mice bearing NB tumors as compared to MDSCs isolated from non-tumor-bearing mice. The increased expression of BTK correlates with a poor relapse-free survival probability of NB patients. Moreover, treatment of NB-tumor-bearing mice with ibrutinib resulted in a significant reduction of MDSCs accompanied by a decrease in immunosuppressive functions of MDSCs. Ibrutinib-treatment further enhanced the antitumor effect of anti-PDL1 checkpoint blockade in a mouse model of NB. These studies suggest that ibrutinib-induced suppression of MDSC function could potentiate the efficacy of checkpoint inhibitors and can serve as a promising strategy to treat patients with NB.

## Data Availability

The raw data that support the finding of study will be made available by the corresponding author on request.

## Supplementary Materials

The following are available online at https://www.mdpi.com/2072-6694/13/4/817/s1, Figure S1: Correlation between BTK and CD14 in neuroblastoma cohorts. Figure S2: Gating strategy of B cells and T cells. Figure S3: Gating strategy of macrophages. Figure S4: Effect of ibrutinib on proliferation of NB cells. Material S1: Images of uncropped western blot membranes.
