# Supplementary material for: BTK Inhibition Reverses MDSC-Mediated Immunosuppression and Enhances Response to Anti-PDL1 Therapy in Neuroblastoma
Source: Cancers (Basel). 2021 Feb 16;13(4):817. doi: 10.3390/cancers13040817 (PMC7919651; doi:10.3390/cancers13040817)
Supplement: Supplementary file 1 [file cancers-13-00817-s001.pdf]

cancers

MDPI

Supplementary Materials

BTK Inhibition Reverses MDSC-Mediated Immunosuppression and Enhances Response to Anti-PDL1 Therapy in Neuroblastoma

Mehreen Ishfaq, Timothy Pham, Cooper Beaman, Pablo Tamayo, Alice L. Yu and Shweta Joshi

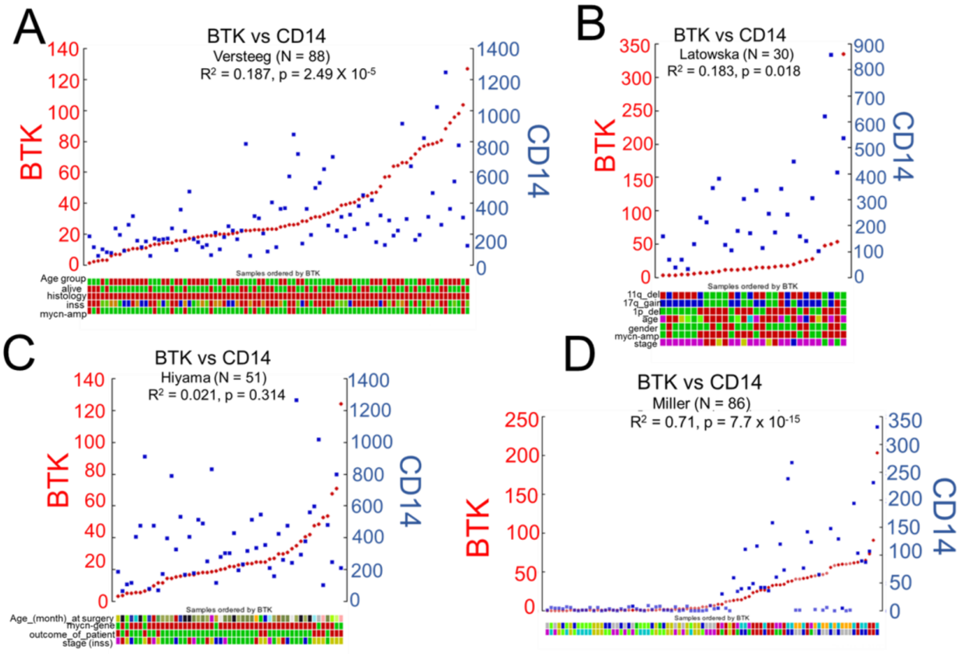

Figure S1. Correlation between BTK and CD14 in neuroblastoma cohorts: **A**–**D**. Positive correlation between BTK and CD14 expression in neuroblastoma cohorts, Versteeg n = 88 (**A**); Latowska n = 30 (**B**); Hiyama n = 51 (**C**) and benign neurofibroma (Miller, n = 86) (**D**).

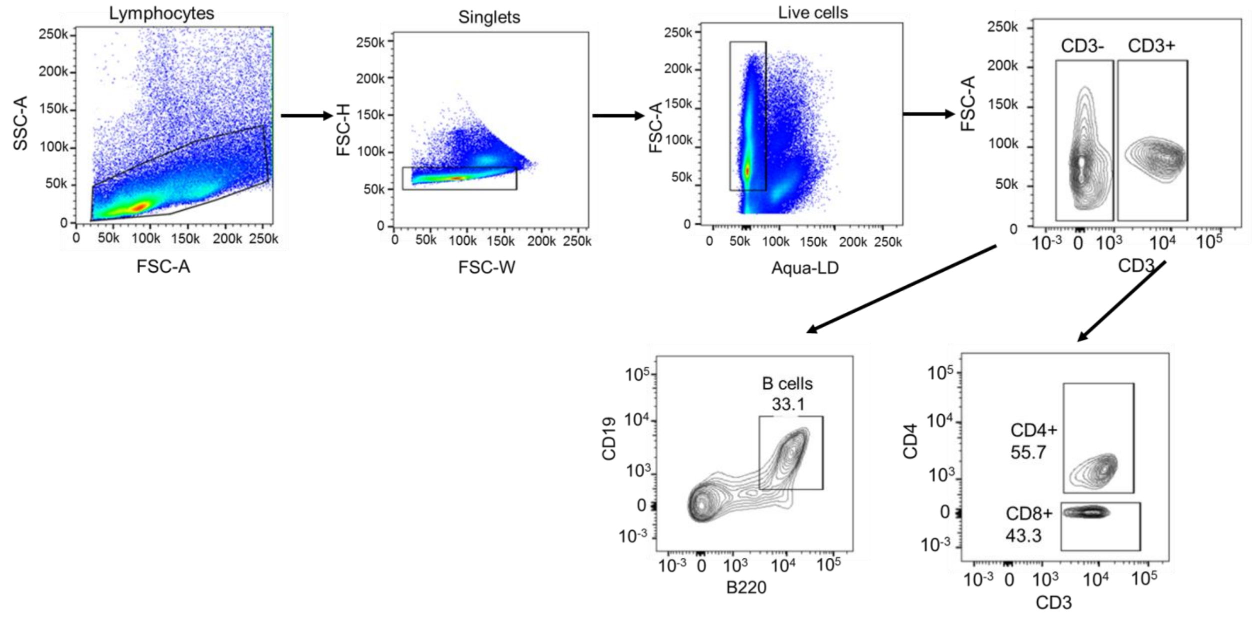

Figure S2. Gating strategy of B cells and T cells.

Fig. S3

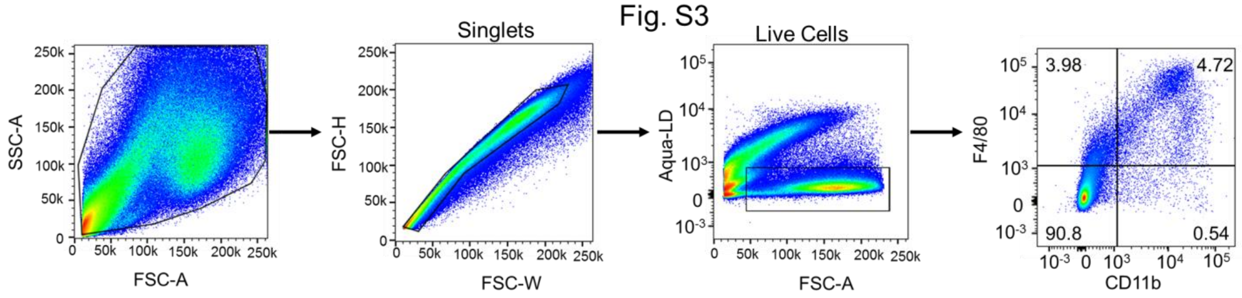

Figure S3. Gating strategy of macrophages.

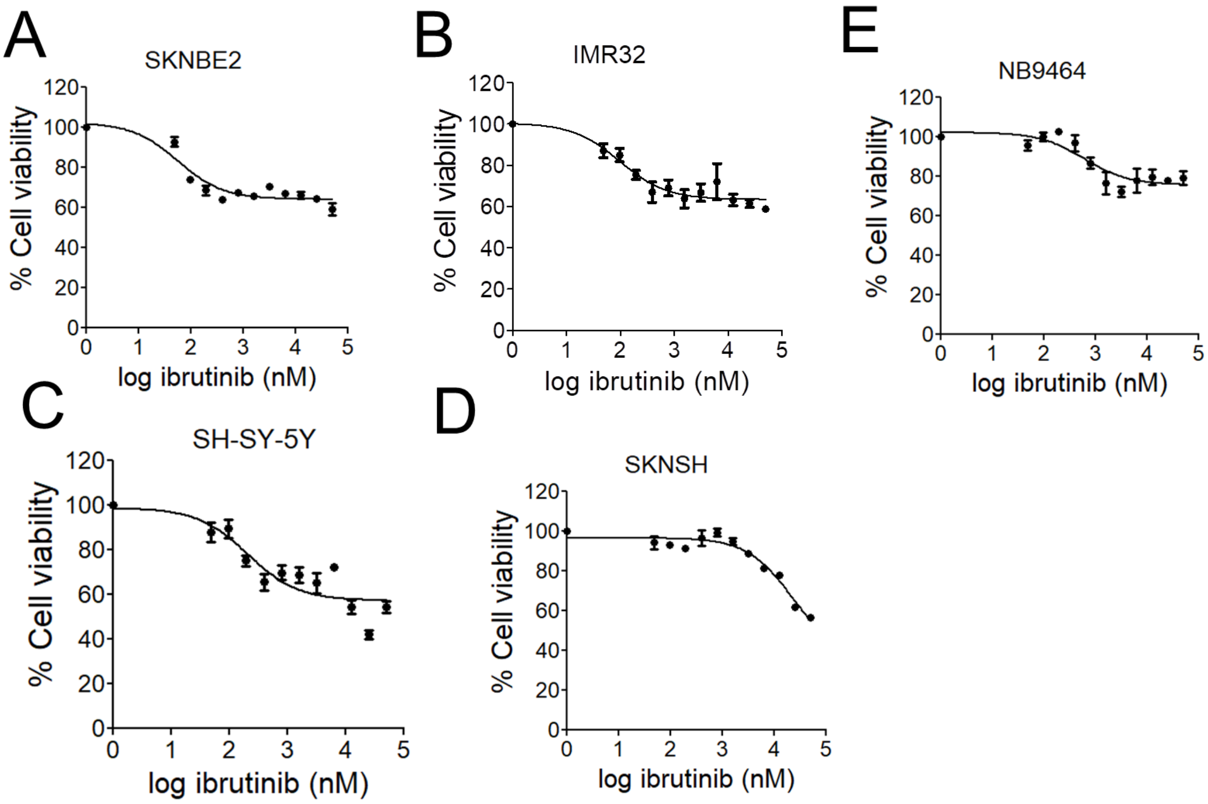

Figure S4. Effect of ibrutinib on proliferation of NB cells: **A–E**. Cell viability assay was performed on SKNBE2, IMR32, SH-SY-5Y, SKNSH and NB9464 NB cells.

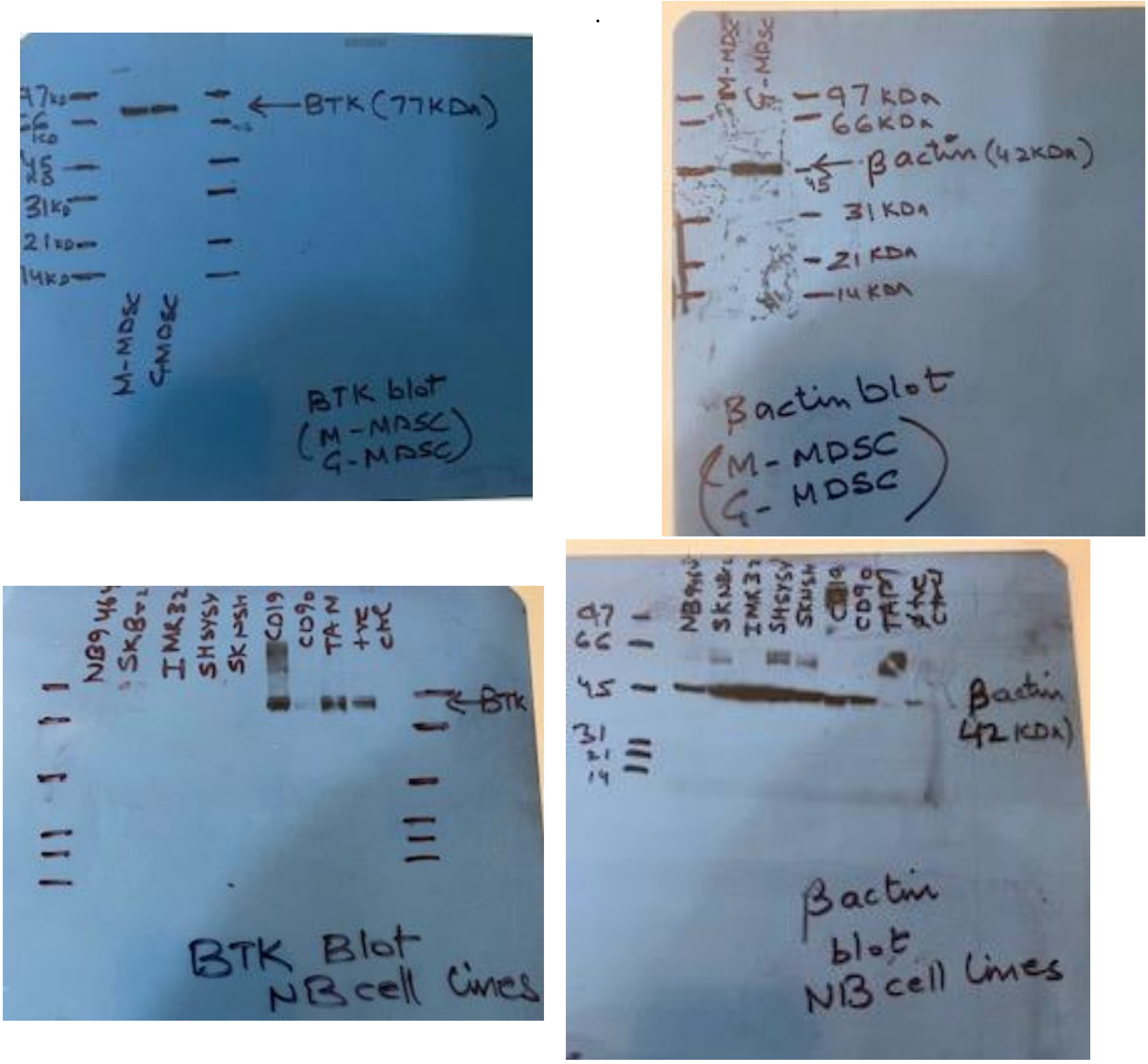

Material S1: Western blot full membrane uncropped images. Uncropped blots related to Figs. 2E and F.
